# Mosquito abundance in relation to extremely high temperatures in urban and rural areas of Incheon Metropolitan City, South Korea from 2015 to 2020: an observational study

**DOI:** 10.1186/s13071-021-05071-z

**Published:** 2021-10-29

**Authors:** Ah-Young Lim, Hae-Kwan Cheong, Yeonseung Chung, Kisung Sim, Jong-Hun Kim

**Affiliations:** 1 Department of Social and Preventive Medicine, Sungkyunkwan University School of Medicine, Suwon, Gyeonggi-do Republic of Korea; 2 Department of Mathematical Sciences, Korea Advanced Institute of Science and Technology, Daejeon, Republic of Korea

**Keywords:** Mosquito-borne diseases, Mosquito population density, Climate change, *Anopheles*, *Aedes*, *Culex*

## Abstract

**Background:**

Despite concerns regarding increasingly frequent and intense heat waves due to global warming, there is still a lack of information on the effects of extremely high temperatures on the adult abundance of mosquito species that are known to transmit vector-borne diseases. This study aimed to evaluate the effects of extremely high temperatures on the abundance of mosquitoes by analyzing time series data for temperature and mosquito abundance in Incheon Metropolitan City (IMC), Republic of Korea, for the period from 2015 to 2020.

**Methods:**

A generalized linear model with Poisson distribution and overdispersion was used to model the nonlinear association between temperature and mosquito count for the whole study area and for its constituent urban and rural regions. The association parameters were pooled using multivariate meta-regression. The temperature–mosquito abundance curve was estimated from the pooled estimates, and the ambient temperature at which mosquito populations reached maximum abundance (TMA) was estimated using a Monte Carlo simulation method. To quantify the effect of extremely high temperatures on mosquito abundance, we estimated the mosquito abundance ratio (AR) at the 99th temperature percentile (AR_99th_) against the TMA.

**Results:**

*Culex pipiens* was the most common mosquito species (51.7%) in the urban region of the IMC, while mosquitoes of the genus *Aedes* (*Ochlerotatus*) were the most common in the rural region (47.8%). Mosquito abundance reached a maximum at 23.5 °C for *Cx. pipiens* and 26.4 °C for *Aedes vexans*. Exposure to extremely high temperatures reduced the abundance of *Cx. pipiens* mosquitoes {AR_99th_ 0.34 [95% confidence interval (CI) 0.21–0.54]} to a greater extent than that of *Anopheles* spp. [AR_99th_ 0.64 (95% CI 0.40–1.03)]. When stratified by region, *Ae. vexans* and *Ochlerotatus koreicus* mosquitoes showed higher TMA and a smaller reduction in abundance at extreme heat in urban Incheon than in Ganghwa, suggesting that urban mosquitoes can thrive at extremely high temperatures as they adapt to urban thermal environments.

**Conclusions:**

We confirmed that the temperature-related abundance of the adult mosquitoes was species and location specific. Tailoring measures for mosquito prevention and control according to mosquito species and anticipated extreme temperature conditions would help to improve the effectiveness of mosquito-borne disease control programs.

**Graphical abstract:**

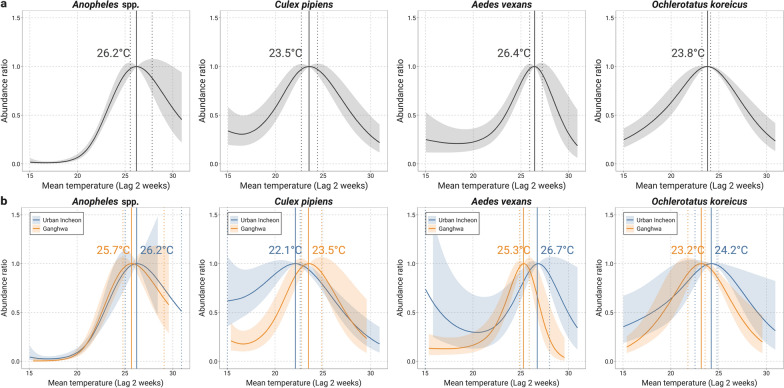

**Supplementary Information:**

The online version contains supplementary material available at 10.1186/s13071-021-05071-z.

## Background

There is a global consensus that climate change has accelerated during the past decades, and there is abundant up-to-date, definitive scientific evidence for global warming and extreme weather events. A rapidly growing body of evidence supports the hypothesis that climate change has already promoted the emergence and re-emergence of vector-borne diseases (VBDs) by altering the life cycles of pathogens and vectors, expanding their geographical distributions, and changing the dynamics of disease transmission [1, 2].

Mosquito vectors are sensitive to climate change. Climatic factors, including temperature and rainfall, influence various aspects of mosquito population dynamics. For example, increasing temperatures are related to increased rates of survival, development, and feeding activity of mosquitoes, which lengthen the transmission seasons of the diseases that they carry [1–3]. Although warm and humid climates are positively associated with mosquito abundance in general, the effect of extremely high temperatures on the life cycle of mosquitoes has been shown to be complex and non-linear, with poor survival and development rates of larvae and feeding behavior of adult mosquitoes after a certain threshold temperature [1, 4–6].

Owing to the temperature-dependent traits of mosquitoes, the incidence of VBDs in endemic areas is projected to increase, and the geographic distribution of VBDs is expected to expand into higher latitudes as a result of global warming. Previous studies have suggested that the spatial distribution of malaria, which is transmitted by *Anopheles* mosquitoes, has shifted toward higher altitudes [7], and that the length of its transmission season has increased in highland regions [8, 9]. Modeling studies carried out in China and Canada have shown that the geographical range of *Culex*, which is responsible for the transmission of West Nile fever and Japanese encephalitis virus, will shift northward under future climate scenarios [10, 11]. Species of the genus *Aedes* that are vectors of dengue and Zika viruses are also projected to spread globally and cause local outbreaks of these diseases in non-endemic areas [12, 13].

Despite growing interest in the implications of climate change on VBD prevention and control, the temperature-dependent population dynamics of mosquitoes are still poorly understood, particularly in terms of their exposure to extreme summer heat waves [1, 4, 6]. It is generally agreed that extreme weather conditions, such as heat waves, flooding, and drought, can have an immediate effect on the risk of VBDs through their deleterious effects on climatic conditions and soil habitats suitable for mosquito proliferation [14, 15], although it remains unclear how heat waves affect mosquito abundance under current conditions [6]. To date, only two studies, carried out in subtropical climates, have reported an association between heat waves and significantly lower abundances of *Aedes albopictus* mosquitoes [16] and *Aedes aegypti* pupae [17]. In addition, information on mosquito growth and survival at high temperatures has relied largely on laboratory findings [3, 18, 19]. Hence, there are many unknowns regarding how mosquitoes respond and adapt to temperature fluctuations in the field.

Therefore, this study aimed to investigate the association between temperature and mosquito abundance by analyzing weekly time series data for temperature and mosquito counts in Incheon Metropolitan City (IMC), Republic of Korea (hereafter ‘Korea’), from 2015 to 2020. In particular, we investigated the impact of extremely high temperature on mosquito abundance based on the observed association between temperature and mosquito abundance. In addition, we stratified our analysis by urban and rural area.

## Methods

### Study area

**Fig. 1:**
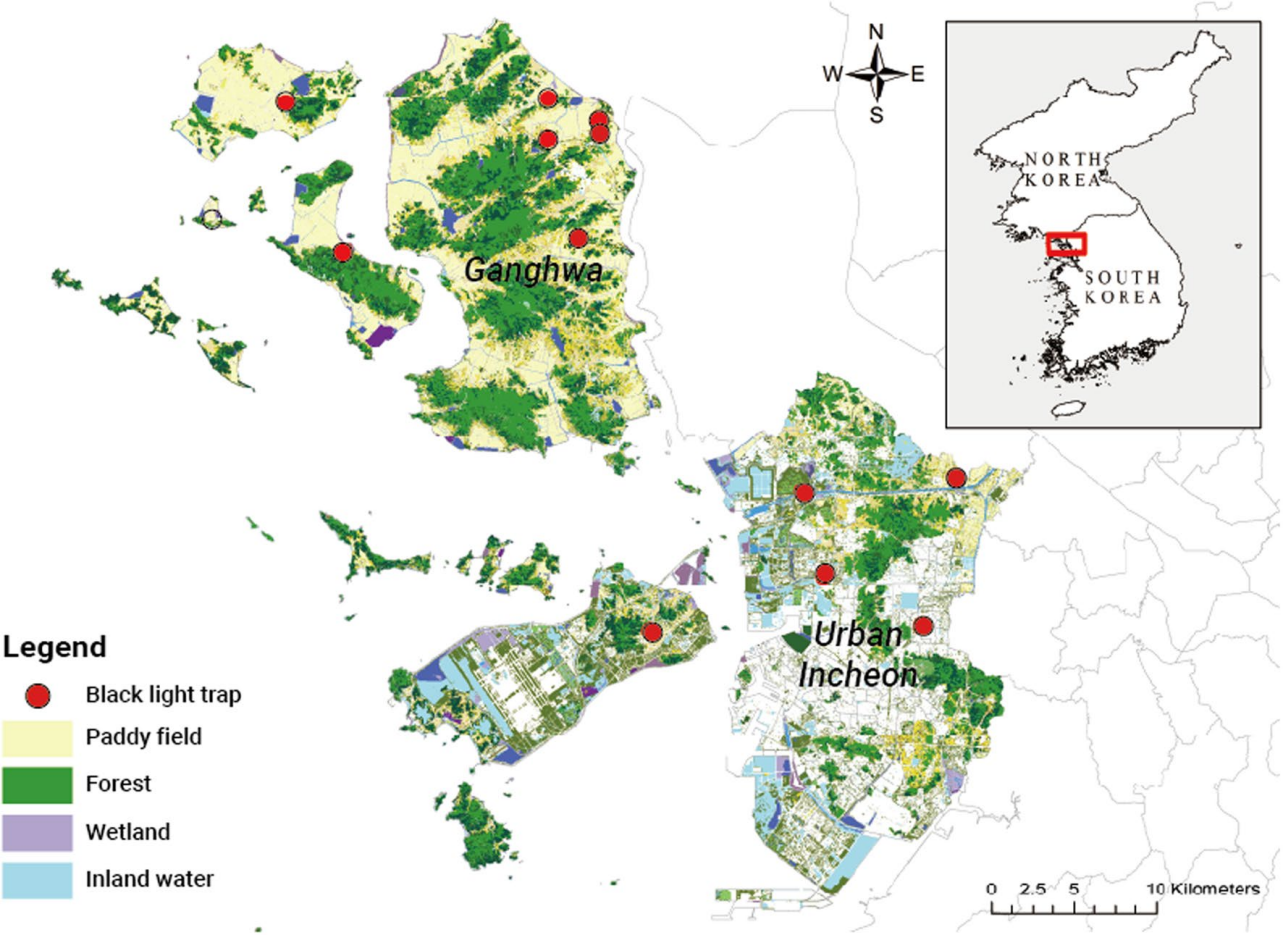
Map of the study area and mosquito monitoring stations in Incheon Metropolitan City

IMC is located on the northwestern coast of Korea (37°27′N, 126°42′E) and borders Seoul Metropolitan City and Gyeonggi-do Province in the east and a demilitarized zone in the north. IMC is classified and managed as a high-risk area for malaria along with other border areas including Gyeonggi-do and Gangwon-do [20]. We divided the study area of IMC into two regions: Ganghwa (rural), where most of the land is covered by forests and rice paddies; and urban Incheon, the remaining, urban area (Fig. 1). The entire study area lies in the temperate zone with four seasons clearly distinguished by temperature rather than rainfall. In total, 12 mosquito collection sites were installed throughout the city between 2015 and 2020 by the city authority. The locations of the 12 monitoring sites (five sites in urban Incheon and seven sites in Ganghwa) are shown in Fig. 1.

Weekly mosquito data were obtained from the Mosquito Surveillance Program of the IMC Institute of Public Health and Environment. From April to October of each year from 2015 to 2020, observational monitoring was performed to investigate the temporal and spatial distribution of vector mosquitoes and predict the local transmission risk of VBDs. Black light traps were used to collect adult mosquitoes for 24 h each day of the week at the 12 monitoring sites (one trap per site) [21]. Female adult mosquitoes were morphologically identified to three main genera [*Anopheles*, *Aedes *(*Ochlerotatus*), and* Culex*] and 16 species (Additional file 1: Table S1), and their counts were recorded in a standardized database format.

Daily climate data were obtained from the Open Weather Data Portal (http://data.kma.go.kr) of the Korean Meteorological Administration. Climatic variables including daily mean, maximum, and minimum temperature (degrees Celsius) and precipitation (millimeters) were summarized as weekly mean temperature and weekly cumulative precipitation for use in the statistical analysis.

Two meteorological observatory stations located in Ganghwa and urban Incheon were selected and the climate data obtained from these were combined with those from seven and five mosquito monitoring sites in Ganghwa and urban Incheon, respectively. To take into account the life cycle of the mosquitoes, i.e., the average number of days from egg hatch to adult emergence, the weekly mean temperature and weekly cumulative precipitation of the 2 weeks prior to the week of mosquito collection were used in the model [22]. Data collected on days with temperatures below 15 °C were removed from the statistical analysis due to the presence of excessive zero mosquito counts.

### Statistical analysis

Weekly data for the number of mosquitoes collected and climatic variables were summarized as the means and SDs for Ganghwa and urban Incheon. The mean differences in climatic variables and mosquito counts between the two regions were tested using Student’s *t*-test.

A two-stage meta-analysis was used to estimate the temperature–mosquito association for the two regions. In the first stage, we separately modeled the association between temperature and mosquito abundance for each collection site. A generalized linear model with Poisson distribution and overdispersion was used to model the nonlinear association between temperature and mosquito count for each species. Let *Y*_*t*_ be the collected mosquito count in week *t*; then the model formula is as follows:

$$Y_{t} \sim {\text{Quasi}}{-}{\text{Poisson}}(\mu_{t} )$$

1 $${\text{Log}}\left( {\mu_{t} } \right) = \alpha + {\text{ns}}\left( {T_{{{\text{mean}}}} } \right) + {\text{ns}}\left( {P_{{{\text{cumulative}}}} } \right) + {\text{ns}}\left( {\text{time trend}} \right)$$

where *μ*_*t*_ is the mean mosquito count at week *t*, *T*_mean_ is weekly mean ambient temperature with a lag of 2 weeks, *P*_cumulative_ is weekly cumulative precipitation with a lag of 2 weeks, and ns is the natural cubic spline for the flexible function describing the nonlinear effects of *T*_mean_, *P*_cumulative_, and time trend. The *df* for the ns(*T*_mean_) were determined based on model selection using the quasi-Akaike information criterion [23]. A *df* of 3 with the knots located at the 50th and 90th percentiles of observed temperature for *Aedes vexans*, 10th and 50th percentiles for *Culex pipiens* and *Anopheles* spp*.*, and 33rd and 66th percentiles for *Ochlerotatus koreicus*, were selected as the optimal model (Additional file 2: Table S1–S4). We adjusted for the long-term trend with 4 *df* per year.

In the second stage, the estimated basis coefficients for ns(*T*_mean_), which correspond to the parameter representing the temperature–mosquito association, were extracted from the fitted model (Eq. 1) along with its SE matrix. These association parameters were then pooled using multivariate meta-regression [24]. Using the pooled estimate, the temperature–mosquito abundance curve was estimated, and the threshold temperature at which the mosquito populations reached the maximum abundance (TMA) was estimated using a Monte Carlo simulation method [25]. The 50th percentile of the Monte Carlo samples was used as a point estimate, and 2.5–97.5th percentiles were used as interval estimates.

To quantify the effect of extremely high temperatures on mosquito abundance, we estimated the mean mosquito abundance ratio (AR) for the 99th temperature percentile (*T*_99th_) for each region versus TMA. We refer to this ratio as the AR for the *T*_99th_ (AR_99th_).

A *P-*value of less than 0.05 was considered significant when interpreting the results. R (version 4.0.3) for Windows was used for all the statistical analyses.

## Results

In total, 80,985 and 2,63,281 mosquitoes were collected from the 12 monitoring sites in urban Incheon and Ganghwa, respectively, between 2015 and 2020. The mean (SD) number of mosquitoes collected per week was 206.0 (607.0) in Ganghwa and 88.5 (136.0) in urban Incheon, about twice as many as in Ganghwa (Table 1). The weekly average mosquito count for the genus *Anopheles* [urban Incheon 15.8 (60.4), Ganghwa 72.1 (254.0)] and *Aedes* (*Ochlerotatus*) [urban Incheon 24.8 (81.8), Ganghwa 98.2 (427.0)] was higher in Ganghwa than in urban Incheon, and the opposite pattern was observed for the genus *Culex* [urban Incheon 45.8 (62.3), Ganghwa 27.2 (54.8)]. The weekly mean temperature was significantly higher in urban Incheon (19.9 °C) than in Ganghwa (19.0 °C) [*t*-test, *t*_(1969.8)_  =  4.2347, *P* <  0.0001]. The weekly average precipitation was 28.9 mm and 26.6 mm in Ganghwa and in urban Incheon, respectively, but the difference was not statistically significant [*t*-test, *t*_(2102.2)_  =  − 1.2639, *P* =  0.2064]. The relative humidity was 74.0% and 70.8% in urban Incheon and Ganghwa, respectively.

**Table 1:** Descriptive statistics of weekly climate variables and mosquito data, 2015–2020 (April–October)

Category	Variables	Area	Mean	SD	Min.	Median	Max.
Climatic variables	Mean temperature (℃)	Urban Incheon	19.9	5.4	7.5	20.9	31.0
		Ganghwa	19.0	5.4	6.9	19.7	29.6
	Minimum temperature (℃)	Urban Incheon	16.8	5.8	4.4	17.6	27.7
		Ganghwa	14.4	6.2	1.2	14.9	25.2
	Maximum temperature (℃)	Urban Incheon	23.7	5.1	11.2	24.7	34.9
		Ganghwa	23.9	4.9	12.0	24.9	33.8
	Precipitation (mm)	Urban Incheon	26.6	40.2	0.0	9.4	258.0
		Ganghwa	28.9	45.8	0.0	7.6	246.0
	Relative humidity (%)	Urban Incheon	74.0	11.3	51.5	74.1	98.4
		Ganghwa	70.8	9.3	45.9	71.0	94.4
Mosquito abundance	Total mosquitoes	Urban Incheon	88.5	136.0	0.0	44.0	1536.0
		Ganghwa	206.0	607.0	0.0	43.0	11,088.0
	Genus *Anopheles*	Urban Incheon	15.8	60.4	0.0	1.0	1043.0
		Ganghwa	72.1	254.0	0.0	3.0	4533.0
	Genus *Aedes *(*Ochlerotatus*)	Urban Incheon	24.8	81.8	0.0	4.0	1519.0
		Ganghwa	98.2	427.0	0.0	12.0	9556.0
	Genus *Culex*	Urban Incheon	45.8	62.3	0.0	26.0	545.0
		Ganghwa	27.2	54.8	0.0	9.0	620.0

**Fig. 2:**
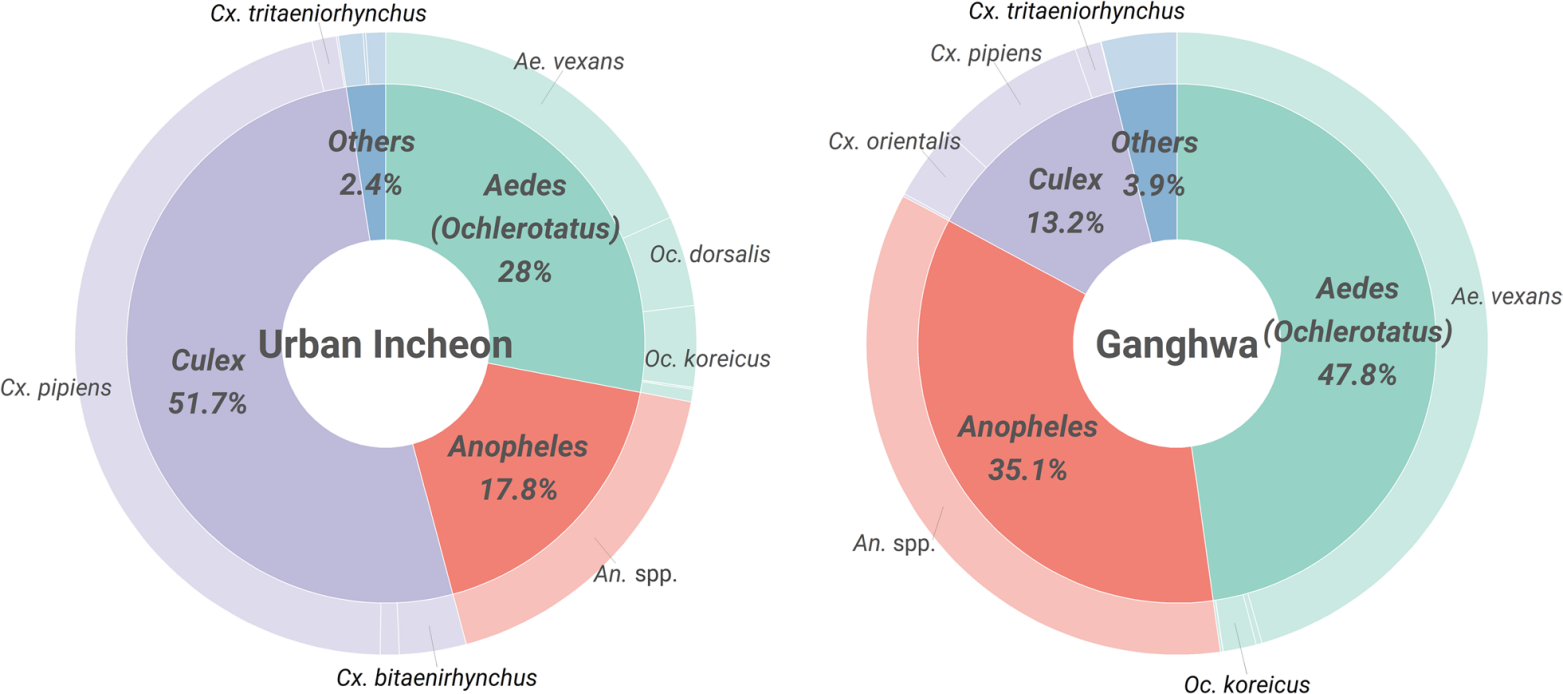
Species distribution of mosquitoes collected in urban Incheon and Ganghwa from 2015 to 2020

There was a difference between urban Incheon and Ganghwa in the species distribution of the mosquitoes collected (Fig. 2). For urban Incheon, almost half of the mosquitoes collected were classified as belonging to the genus *Culex* (51.7%), and most of these were *Culex pipiens*. The genera *Aedes* (*Ochlerotatus*) and *Anopheles* accounted for 28.0% and 17.8%, respectively, of the mosquitoes collected in urban Incheon. In contrast, *Aedes* (*Ochlerotatus*) mosquitoes accounted for more than half (47.8%) of the total mosquitoes collected in Ganghwa, followed by the genera *Anopheles* and *Culex*, which accounted for 35.1% and 13.2%, respectively. For both regions, *Ae. vexans* accounted for most of the *Aedes* (*Ochlerotatus*) mosquitoes collected, followed by *Oc. koreicus*.

**Fig. 3:**
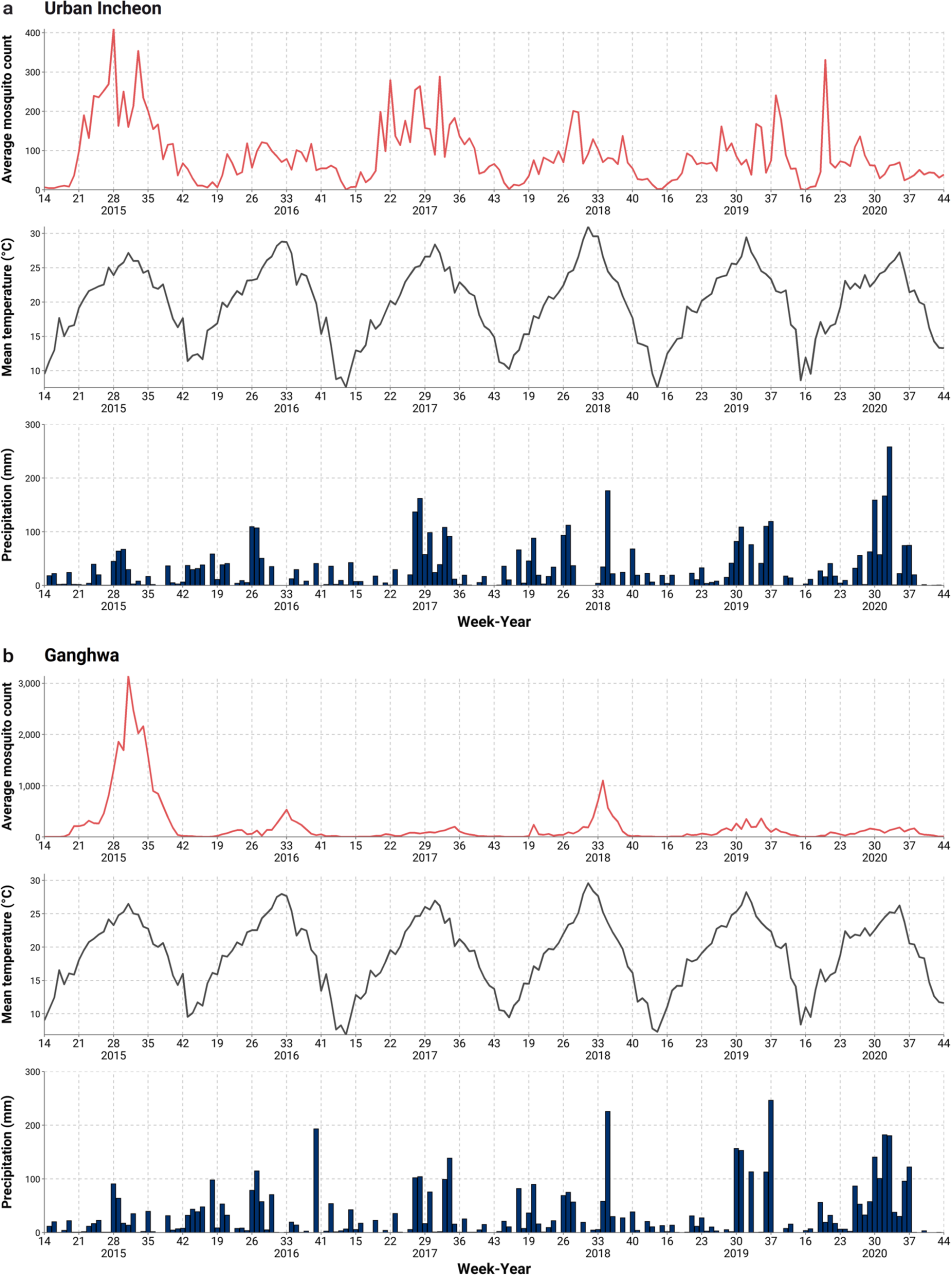
Time series plot of weekly mosquito abundance, temperature, and precipitation in** a** urban Incheon and** b** Ganghwa, 2015–2020

In general, the average mosquito count tended to peak in summer, particularly between weeks 28 and 35 (Fig. 3). The average mosquito abundance peaked at week 28 of 2015 in both urban Incheon and Ganghwa and in week 34 of 2018 in Ganghwa. Throughout the period 2015–2020, the mean temperature peak was 30.1 °C and 29.9 °C, in week 31 of 2018, in urban Incheon and Ganghwa, respectively. The *T*_99th_ was 29.6 °C for all regions, and 29.6 °C and 28.4 °C for urban Incheon and Ganghwa, respectively.

**Fig. 4:**
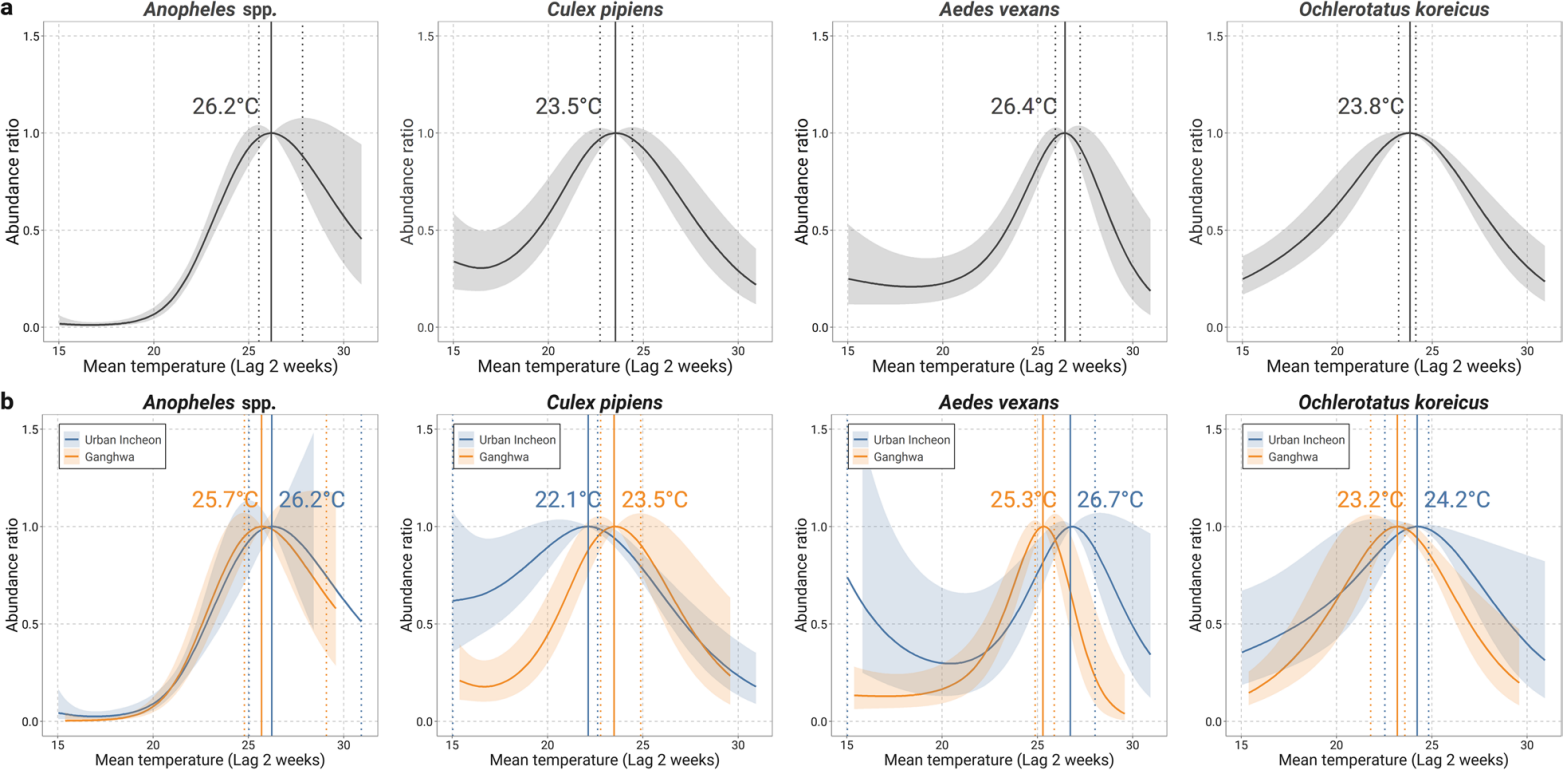
The association between temperature and mosquito abundance by mosquito species and by region. **a** Entire study area, **b** urban Incheon and Ganghwa

We estimated the TMA by mosquito species and mosquito AR for the range of observed temperatures compared with TMA (Fig. 4a). The TMA varied by mosquito species, with the highest temperature of 26.4 ℃ (2.5–97.5th percentile 25.9–27.2 ℃) for *Ae. vexans* and the lowest of 23.5 ℃ (2.5–97.5th percentile 22.7–24.4 ℃) for *Cx. pipiens*. Maximum abundance was at 26.2 ℃ (2.5–97.5th percentile 25.5–27.8 ℃) for *Anopheles* species and at 23.8 °C (2.5–97.5th percentile 23.2–24.1 °C) for *Oc. koreicus*. Although the AR curves generally had an inverted U-shape, the shapes were slightly different among the species. While the number of *Oc. koreicus* and *Cx. pipiens* steadily increased with an increase in temperature above 15 °C, the number of *Ae. vexans* and mosquitoes of the genus *Anopheles* remained roughly the same when the temperature increased up to 20.0 °C, but began to increase rapidly at temperatures above 20.0 °C. When stratified by region, the overall trend was similar to that for the entire area, but the TMA by mosquito species was higher in urban Incheon than in Ganghwa, except for *Cx. pipiens* (Fig. 4b).

**Fig. 5:**
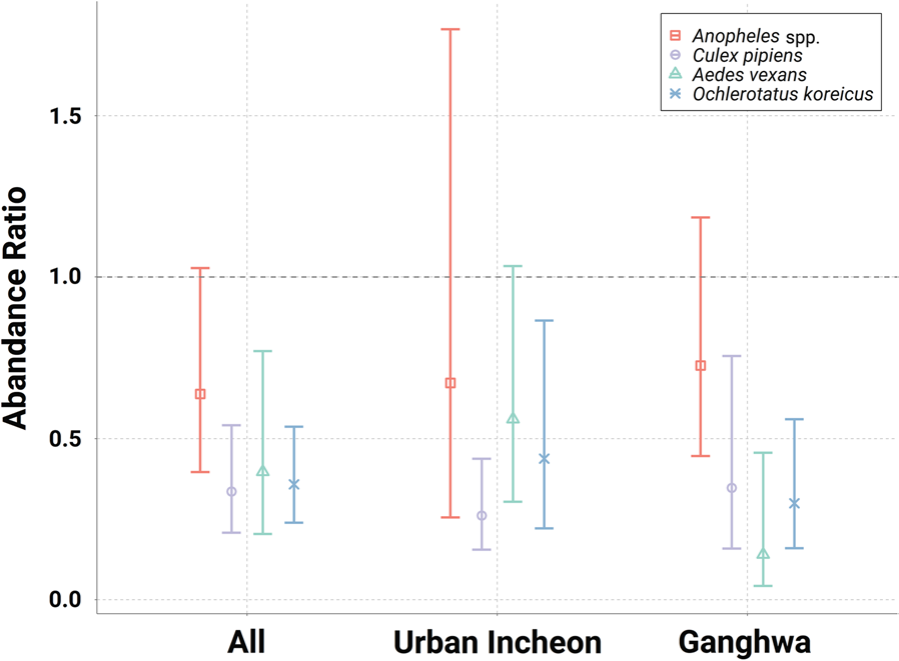
The abundance ratio for the 99th percentile temperature versus the temperature at which the mosquito population reached maximum abundance (dashed horizontal line)

Extreme heat was associated with reductions in *Cx. pipiens* [AR_99th_ 0.34 (95% CI 0.21–0.54)], *Oc. koreicus* [AR_99th_ 0.36 (95% CI 0.24–0.54)], and *Ae. vexans* [AR_99th_ 0.40 (95% CI 0.200.77)], but the decline in the number of individuals of the genus *Anopheles* [AR_99th_ 0.64 (95% CI 0.40–1.03)] was non-significant. (Fig. 5). When stratified by region, the reductions in mosquito abundance at *T*_99th_ was statistically significant in Ganghwa for all mosquito species except for *Anopheles* spp. In particular, *Ae. vexans* and *Oc. koreicus* showed more pronounced reductions in Ganghwa than in urban Incheon: the AR_99th_ of *Ae. vexans* was 0.14 (95% CI 0.04–0.46) in Ganghwa and 0.56 (95% CI 0.30–1.03) in urban Incheon, the AR_99th_ of *Oc. koreicus* was 0.30 (95% CI 0.16–0.56) in Ganghwa and 0.44 (95% CI 0.22–0.86) in urban Incheon (Fig. 5).

## Discussion

We investigated the effects of extremely hot weather on mosquito abundance based on weekly time series data collected over a 6-year period in the field. We were able to confirm that the different mosquito species had different TMA values. In addition, we found that the impact of extremely high temperatures on mosquito abundance differed between the two regions, with a more pronounced reduction of mosquito abundance for rural Ganghwa than for urban Incheon.

In general, we could confirm a non-linear association between temperature and mosquito density, indicating that mosquito abundance increases with an increase in air temperature and declines after reaching a threshold temperature. This was consistent with previous laboratory-based studies on the effects of temperature on the development and survival of vector mosquitoes such as *Aedes aegypti* [26], *Aedes albopictus* [3], and *Anopheles gambiae* [27, 28]. We also found that the threshold temperatures at which the number of mosquitoes began to decrease varied depending on the vector species, and ranged from 23.5 °C for *Cx. pipiens* to 26.4 °C for *Ae. vexans*. A previous study [29] estimated the empirical mean temperature for maximum mosquito abundance using daily mosquito monitoring data and found threshold temperatures of 22.6 °C for *Cx. pipiens* and 22.4 °C for the *Anopheles hyrcanus* group, which are lower than our estimates. Another study carried out in Singapore reported that the increase in adult *Culex* mosquito abundance plateaued at a mean temperature of 26.8 °C [30]. The range of temperature optima for adult mosquito survival in the present study was similar to experimental findings from previous studies, which showed that the best thermal performance when the temperature was between 25 and 30 °C varied by vector species [3, 18, 19].

We could confirm that most vector mosquitoes in the urban areas had a higher TMA and AR_99th_ than those in the rural areas, which indicates that urban mosquitoes have the ability to survive at high temperatures and that the reduction in their abundance is small even at extremely high temperatures. These results agree with previous findings on the adaptation of mosquitoes to local temperatures [31] and to urban environments, e.g., *Aedes aegypti* [32, 33], *Aedes albopictus* [34], and *Anopheles sinensis* [35]. The finding that mosquito abundance at an extremely high temperature had decreased by more than half in Ganghwa but showed a less pronounced decrease in urban Incheon is consistent with adaptation to the local thermal environment. Higher average temperatures and greater temperature fluctuations in urban areas may contribute to urban mosquitoes being less sensitive to temperature changes [31, 36, 37]. Conversely, as the temperature variation in rural areas is relatively small, a small increase in temperature may have contributed significantly to the decrease in mosquito density in Ganghwa.

The impacts of climate change and increasing temperatures on vector population dynamics may be more complex than previously acknowledged. According to the Korea Meteorological Administration, the average temperature on the Korean Peninsula will increase by more than 2 ℃ by the end of the twenty-first century under Representative Concentration Pathway 4.5 and by more than 4 ℃ under Representative Concentration Pathway 8.5 [38]. It is generally agreed that the distribution of vectors will expand to higher latitudes and that the temperature range, presently 23–26 °C, at which mosquito density is maximized will extend in the long term with further increases in global temperature. Concurrently, with increasing urbanization and more frequent and intense heat waves, region- and species-specific changes in mosquito population dynamics are expected to be observed more commonly in the future.

This study had several limitations. First, we did not adjust for non-climatic drivers, such as agricultural practices and land use change, which are widely known to affect the abundance of adult mosquitoes [1, 2]. However, a comparison of land use in 2015 and 2019 in IMC showed that there was no significant land use change in the region, except in urban Incheon. Second, the genus *Anopheles* was not classified into separate species and individuals of this taxon were evaluated cumulatively. Six *Anopheles* species have been reported for Korea and comprise a species complex called *Anopheles sinensis* sensu lato: *Anopheles sinensis* sensu stricto, *Anopheles lesteri*, *Anopheles pullus*, *Anopheles sineroides*, *Anopheles kleini*, and *Anopheles belenrae* [39]. As molecular methods are required to distinguish these species because it is difficult to achieve this morphologically, species identification of *Anopheles* spp. is rarely conducted during real-time vector monitoring in Korea [39]. Oh et al. [40] reported that *An. sinensis* is the most abundant of these species in Ganghwa, followed by *An. belenrae* and *An. pullus*. Third, because climate data from two weather stations representing urban Incheon and Ganghwa were used, the differences in climate between the mosquito collection sites may not be fully reflected in the results. However, as the maximum distance between the weather stations and the mosquito collection sites was 18 km, the differences in climate data are likely to be insignificant. Fourth, mosquito collection and species identification were conducted on a weekly basis, which limited our ability to identify the effects of daily variations in temperature on mosquito density.

## Conclusions

We could confirm that there is a non-linear association between temperature and mosquito abundance, and showed that the shape of the temperature–mosquito abundance curve, and the threshold temperature at which mosquito abundance reached a maximum, varied by mosquito species. In particular, there was a difference in the reduction in the abundance of mosquitoes between urban Incheon and Ganghwa at extremely high temperatures, which may be attributable to differences between the thermal adaptation of mosquitoes inhabiting urban and rural areas. Therefore, tailoring prevention and control measures according to mosquito species and anticipated extreme temperature conditions would help to improve the effectiveness of mosquito-borne disease control programs.

## Supplementary Information

**Additional file 1: Table S1.** List of mosquito species collected by the Incheon Metropolitan City Institute of Public Health and Environment, 2015–2020.

**Additional file 2: Table S1.** Quasi-Akaike information criterion values of models for *Anopheles *spp. **Table S2.** Quasi-Akaike information criterion values of models for *Culex pipiens*. **Table S3.** Quasi-Akaike information criterion values of models for *Aedes vexans*. **Table S4.** Quasi-Akaike information criterion values of models for *Ochlerotatus koreicus*.

## Data Availability

The datasets used during and/or analyzed during the current study are available from the corresponding author on reasonable request.

## Abbreviations

AR: Abundance ratio
IMC: Incheon Metropolitan City
ns: Natural cubic spline
*P*_cumulative_: Weekly cumulative precipitation with a lag of 2 weeks
*T*_mean_: Weekly mean ambient temperature with a lag of 2 weeks
TMA: Temperature at which mosquito populations reached maximum abundance
VBD: Vector-borne disease
