# Supplementary material for: Mosquito abundance in relation to extremely high temperatures in urban and rural areas of Incheon Metropolitan City, South Korea from 2015 to 2020: an observational study
Source: Parasit Vectors. 2021 Oct 29;14:559. doi: 10.1186/s13071-021-05071-z (PMC8555308; doi:10.1186/s13071-021-05071-z)
Supplement: Supplementary file 1 — Additional file 1: Table S1. List of mosquito species collected by the Incheon Metropolitan City Institute of Public Health and Environment, 2015–2020. [file 13071_2021_5071_MOESM1_ESM.docx]

**Additional file 1**

**Table S1.** List of mosquito species collected by the Incheon Metropolitan City Institute of Public Health and Environment, 2015-2020.

| **Genus** | **Species** |  |
| --- | --- | --- |
| *Anopheles* | *Anopheles* spp. | |
| *Culex* | *Culex inatomii* | |
|  | *Culex bitaeniorhynchus* | |
|  | *Culex orientalis* | |
|  | *Culex pipiens* | |
|  | *Culex tritaeniorhynchus* | |
|  | *Culex vagans* | |
| *Aedes (Ochlerotatus)* | *Aedes albopictus* | |
|  | *Aedes vexans* | |
|  | *Ochlerotatus* *koreicus* | |
|  | *Ochlerotatus* togoi | |
|  | *Ochlerotatus dorsalis* | |
| Others | *Armigeres subalbatus* | |
|  | *Coquillettidia ochracea* | |
|  | *Mansonia uniformis* | |
