# Supplementary material for: Mosquito abundance in relation to extremely high temperatures in urban and rural areas of Incheon Metropolitan City, South Korea from 2015 to 2020: an observational study
Source: Parasit Vectors. 2021 Oct 29;14:559. doi: 10.1186/s13071-021-05071-z (PMC8555308; doi:10.1186/s13071-021-05071-z)
Supplement: Supplementary file 2 — Additional file 2: Table S1. Quasi-Akaike information criterion values of models for Anopheles spp. Table S2. Quasi-Akaike information criterion values of models for Culex pipiens. Table S3. Quasi-Akaike information criterion values of models for Aedes vexans. Table S4. Quasi-Akaike information criterion values of models for Ochlerotatus koreicus. [file 13071_2021_5071_MOESM2_ESM.docx]

**Additional file 2**

**Table S1.** Quasi-Akaike information criterion values of models for *Anopheles* spp.

| Monitoring sites | Location of knots | | |
| --- | --- | --- | --- |
|  | 10^th^ and 50^th^ percentiles | 50^th^ and 90^th^ percentiles | 33^th^ and 66^th^ percentiles |
| Daesan-ri, Ganghwa-eup | 13,071.8 | 38,834.3 | 153,664.4 |
| Wolgot-ri, Ganghwa-eup | 17,433.4 | 16,790.6 | 16,650.7 |
| Daeryoung-ri, Gyodong-myeon | 22,065.6 | 352,628.7 | 22,206.8 |
| Seogeom-ri, Samsan-myeon | 42,748.4 | 97,055.9 | 56,378.5 |
| Geumwol-ri, Soenwon-myeon | 17,443.2 | 17,451.5 | 17,584.0 |
| Soljeong-ri, Songhae-myeon | 7,657.9 | 8,932.8 | 8,211.8 |
| Sungnoe-ri, Songhae-myeon | 15,098.7 | 13,441.3 | 13,052.9 |
| Seonjuji-dong, Gyeyang-gu | 2,940.1 | 2,934.2 | 2,939.4 |
| Bupyeong-dong, Bupyeong-gu | 503.7 | 512.1 | 506.1 |
| Baekseok-dong, Seo-gu | 1,602.1 | 1,646.6 | 1,630.0 |
| Yeonhui-dong, Seo-gu | 826.8 | 862.5 | 830.1 |
| Unnam-dong, Jung-gu | 13,062.0 | 149,723.3 | 15,599.4 |
| Total | 154,453.7 | 700,813.7 | 309,254.3 |

**Table S2.** Quasi-Akaike information criterion values of models for *Culex pipiens*.

| Monitoring sites | Location of knots | | |
| --- | --- | --- | --- |
|  | 10^th^ and 50^th^ percentile | 50^th^ and 90^th^ percentiles | 33^th^ and 66^th^ percentiles |
| Daesan-ri, Ganghwa-eup | 2,996.3 | 2,979.4 | 3,030.4 |
| Wolgot-ri, Ganghwa-eup | 2,617.0 | 2,668.1 | 2,618.8 |
| Daeryoung-ri, Gyodong-myeon | 3,689.7 | 3,811.7 | 3,750.2 |
| Seogeom-ri, Samsan-myeon | 4,597.6 | 4,743.5 | 4,657.9 |
| Geumwol-ri, Soenwon-myeon | 5,476.9 | 5,545.5 | 5,537.7 |
| Soljeong-ri, Songhae-myeon | 4,926.3 | 4,932.0 | 4,958.4 |
| Sungnoe-ri, Songhae-myeon | 3,766.1 | 3,957.2 | 3,829.8 |
| Seonjuji-dong, Gyeyang-gu | 4,792.7 | 4,865.7 | 4,829.3 |
| Bupyeong-dong, Bupyeong-gu | 4,481.1 | 4,482.0 | 4,512.1 |
| Baekseok-dong, Seo-gu | 10,242.8 | 10,441.8 | 10,364.5 |
| Yeonhui-dong, Seo-gu | 10,522.0 | 10,435.6 | 10,378.7 |
| Unnam-dong, Jung-gu | 5,327.9 | 5,392.0 | 5,367.6 |
| Total | 63,436.2 | 64,254.5 | 63,835.3 |

**Table S3.** Quasi-Akaike information criterion values of models for *Aedes vexans*.

| Monitoring sites | Location of knots | | |
| --- | --- | --- | --- |
|  | 10^th^ and 50^th^ percentiles | 50^th^ and 90^th^ percentiles | 33^th^ and 66^th^ percentiles |
| Daesan-ri, Ganghwa-eup | 31,706.7 | 33,273.7 | 31,901.7 |
| Wolgot-ri, Ganghwa-eup | 121,543.6 | 97,377.7 | 106,520.7 |
| Daeryoung-ri, Gyodong-myeon | 41,922.5 | 38,493.5 | 39,224.2 |
| Seogeom-ri, Samsan-myeon | 23,030.6 | 19,364.3 | 20,879.2 |
| Geumwol-ri, Soenwon-myeon | 16,344.7 | 15,302.9 | 15,553.7 |
| Soljeong-ri, Songhae-myeon | 54,410.2 | 52,595.8 | 52,681.6 |
| Sungnoe-ri, Songhae-myeon | 40,354.7 | 34,410.5 | 33,970.3 |
| Seonjuji-dong, Gyeyang-gu | 15,649.2 | 15,966.6 | 16,110.3 |
| Bupyeong-dong, Bupyeong-gu | 293.6 | 290.1 | 291.1 |
| Baekseok-dong, Seo-gu | 3,390.4 | 3,224.6 | 3,327.4 |
| Yeonhui-dong, Seo-gu | 1,044.6 | 1,031.5 | 1,032.1 |
| Unnam-dong, Jung-gu | 6,340.8 | 6,174.5 | 6,208.8 |
| Total | 356,031.4 | 317,505.7 | 327,700.9 |

**Table S4.** Quasi-Akaike information criterion values of models for *Ochlerotatus koreicus*.

| Monitoring sites | Location of knots | | |
| --- | --- | --- | --- |
|  | 10^th^ and 50^th^ percentiles | 50^th^ and 90^th^ percentiles | 33^th^ and 66^th^ percentiles |
| Daesan-ri, Ganghwa-eup | 1,132.3 | 1,121.1 | 1,125.2 |
| Wolgot-ri, Ganghwa-eup | 1,256.5 | 1,281.9 | 1,261.0 |
| Daeryoung-ri, Gyodong-myeon | 1,219.7 | 1,214.9 | 1,214.0 |
| Seogeom-ri, Samsan-myeon | 1,019.3 | 1,003.0 | 1,001.9 |
| Geumwol-ri, Soenwon-myeon | 1,072.5 | 1,063.2 | 1,068.0 |
| Soljeong-ri, Songhae-myeon | 763.6 | 766.6 | 764.9 |
| Sungnoe-ri, Songhae-myeon | 2,044.1 | 2,046.2 | 2,045.1 |
| Seonjuji-dong, Gyeyang-gu | 1,018.9 | 1,019.8 | 1,016.5 |
| Bupyeong-dong, Bupyeong-gu | 385.1 | 383.9 | 384.4 |
| Baekseok-dong, Seo-gu | 635.1 | 640.9 | 642.4 |
| Yeonhui-dong, Seo-gu | 333.2 | 331.8 | 332.8 |
| Unnam-dong, Jung-gu | 2,025.3 | 1,972.1 | 1,972.5 |
| Total | 12,905.5 | 12,845.5 | 12,828.7 |
